# Circulating tumor DNA predicts clinical benefits of immune checkpoint blockade in HER2-negative patients with advanced gastric cancer

**DOI:** 10.1007/s10120-025-01621-x

**Published:** 2025-05-15

**Authors:** Mei He, Congcong Ji, Zhiwei Li, Shiqing Chen, Jing Gao, Lin Shen, Cheng Zhang

**Affiliations:** 1 Department of Oncology, Shenzhen Key Laboratory of Gastrointestinal Cancer Translational Research, Cancer Institute, Peking University Shenzhen Hospital, Shenzhen-Peking University-Hong Kong University of Science and Technology Medical Center, Shenzhen, 518036 China; 2 Key Laboratory of Carcinogenesis and Translational Research (Ministry of Education/Beijing), Department of Gastrointestinal Oncology, Peking University Cancer Hospital & Institute, Beijing, 100142 China; 3 Department of Clinical and Translational Medicine, 3D Medicines Inc., Shanghai, 201114 China

**Keywords:** Advanced gastric cancer, HER2-negative, Circulating tumor DNA, Liquid biopsy, Immune checkpoint blockade

## Abstract

**Background:**

Immune checkpoint inhibitors (ICIs) are becoming more prominent in the treatment of gastric cancer (GC). However, predictive biomarkers of response to ICIs in HER2-negative patients remain incompletely understood.

**Methods:**

A total of 47 patients diagnosed with HER2-negative advanced GC who underwent ICI regimens were eligible for this study. Plasma samples with paired white blood cells prior to treatments were collected from these 47 patients. Variations of circulating tumor DNA (ctDNA) was evaluated by next-generation sequencing followed by its significance analysis.

**Results:**

A total of 658 somatic mutations involving 203 genes were identified in all ctDNA. Mutations in *MEN1*, *MLH1*, *CEBPA*, *ATR*, *GNAQ*, and *FOXL2* genes were more frequent in responders (*P* < 0.05). Compared with wild-type patients, patients with *CEBPA* or *IRS2* mutations had prolonged median progression-free survival (mPFS, *P* = 0.0056). Patients with co-occurring mutations in *IRS2*/*CEBPA*, *IRS2*/*POLD1*, *TP53*/*PIK3CA*, or *POLD1*/*CEBPA* had longer mPFS compared with others (*P* = 0.003; 0.006; 0.0166; 0.0315; respectively). Both alteration of *CDKN2A* alone and co-mutations with *MSH6* were significantly associated with superior overall survival (OS,* P* = 0.0289; 0.0355; respectively). In addition, higher on-treatment ctDNA concentration or variant allele frequency (VAF) were associated with poorer response (*P* < 0.05). Additionally, the increased molecular alterations of *POLE*, *FGFR2* and *MDC1* seemed to indicate the acquired resistance to ICIs.

**Conclusions:**

Variation signatures captured by ctDNA as well as the kinetics of ctDNA could predict the clinical benefits of ICB in HER2-negative GC patients, which was worth further validated in large cohort.

**Supplementary Information:**

The online version contains supplementary material available at 10.1007/s10120-025-01621-x.

## Introduction

Globally, gastric cancer (GC) remains the fifth most common malignant cancer and the global burden of this malignancy is expected to have a 62% increase by 2040 [1]. Due to the insidious onset and the lack of specific clinical manifestations, most diagnosed patients present at advanced stages with poor prognosis, making it the fourth leading cause of cancer-related mortality [1, 2]. Although the treatment of gastric cancer has gradually developed from traditional surgical resection and systemic chemotherapy to a comprehensive treatment model combining radiotherapy, targeted therapy and immunotherapy, the 5-year survival rate of gastric cancer patients is still unsatisfactory. In addition, the biological behavior of gastric cancer is complex and changeable with high intra and inter-tumor heterogeneity, resulting in poor treatment effects. As a result, there is a significant clinical need to develop sensitive, specific, convenient and real-time biomarkers to reflect the existence, progression and transformation of tumor to better guide treatment options.

Circulating tumor DNA (ctDNA), consisting of cell-free DNA fragments shed by primary and metastatic tumor cells into the plasma, carries tumor-specific genetic features and epigenetic alterations. In comparison to conventional tissue biopsies, ctDNA offers numerous advantages as liquid biopsy analyte. It enables the capture of tumor genomic profiles from minimally invasive blood samples, overcomes tumor heterogeneity, and allows for dynamic monitoring of treatment response and prediction of prognosis [3]. Moreover, ctDNA analysis has demonstrated superior sensitivity in detecting tumor-specific genetic changes than tissue testing [4] and forecasting disease progression earlier than computed tomography scanning [5]. Accumulating evidence has emphasized the potential of ctDNA to predict treatment response, detect and monitor disease progression, evaluate prognosis and adverse effects, identify mechanisms of resistance in gastric/ gastro-oesophageal cancer [4, 6–11].

With increased understanding of the tumor microenvironment and immune targets, immune checkpoint blockade (ICB) has emerged as an exciting treatment strategy across a spectrum of malignancies. Blocking the interaction of immune checkpoints and their ligands by immune checkpoint inhibitors (ICIs) may relieve immune cells from inhibition caused by immune checkpoints, thereby reinvigorating immune cells to exert anti-tumor effects. This includes ICIs targeting programmed death protein-1 (PD-1)/programmed death-ligand 1 (PD-L1) and cytotoxic T lymphocyte antigen 4 (CTLA-4) pathways [12]. The studies of ATTRACTION-02 and KEYNOTE-059 first established the status of ICI monotherapy in patients with third-line and posterior-line advanced GC [13, 14]. Furthermore, results of clinical trials have shown that the combination of ICIs with chemotherapy [15–19], anti-human epidermal growth factor receptor 2 (HER2) targeted therapy [20], and dual ICIs [21–23], may improve the clinical treatment efficiency of patients with advanced GC. For HER2-negative patients, immunotherapy plus chemotherapy has become the recommended first-line standard [24, 25]. Despite the many improvements made in ICI therapy, responses are often limited to a subset of patients. For example, the effect of ICIs on patients with varying PD-L1 expression levels remains debated. Therefore, it is necessary to screen out patients who can benefit from ICI regimens to guide precision treatment.

Potential predictive biomarkers including microsatellite instability (MSI) status, combined positive score (CPS) of PD-L1 expression, tumor mutational burden (TMB) and Epstein-Barr virus (EBV) status have thus far been demonstrated to predict response to immunotherapy [26, 27]. However, most tissue-based GC biomarkers carry the risk of sampling error due to intratumoral heterogeneity, so the development of liquid biopsy-based ctDNA biomarkers is of paramount importance. In this retrospective study, we aimed to evaluate the utility of plasma ctDNA in patients with HER2-negative GC who lacked an effective therapeutic target to determine the mutational profile, assess tumor burden, and identify responders who were likely to benefit from ICI-containing treatments in accordance with ctDNA-based biomarkers.

## Methods

### Study design and patient information

This is a retrospective, single-centre study conducted at Peking University Cancer Hospital. The ctDNA analysis was performed on prospectively collected samples from patients who met the following criteria: (1) age ≥ 18 years; (2) diagnosed with unresectable or metastatic GC; (3) received single-agent or double-agent ICI treatment, in combination with or without chemotherapy; and (4) baseline blood samples were collected for ctDNA sequencing reports prior to ICI treatment.

The patients’ clinical data were collected, including patient demographics, histologic subtypes, Lauren classification, tumor stage, tumor site, plasma Epstein–Barr virus (EBV) status, tissue MSI/HER2/PD-L1 status, therapy regimens, lines of treatment, and the best overall response (BOR). HER2 positivity was assessed using immunohistochemistry (IHC), defined as IHC3 positive or IHC2 positive, and amplified fluorescence in situ hybridization (FISH), defined as a HER2 to chromosome enumeration probe 17 ratio of 2.0 or more. PD-L1 status was determined by IHC, and PD-L1 positivity was defined as a CPS ≥ 1. By referring to computed tomography (CT)-scanned imaging, treatment responses were assessed according to RECIST 1.1 criteria and classified as complete response (CR), partial response (PR), stable disease (SD) and progressive disease (PD). Objective response (OR) was defined as PR or CR.

This study was conducted in accordance with the declaration of Helsinki. These specimens and associated clinical information were approved for experimental applications by the Institutional Ethics Committee, Peking University Cancer Hospital & Institute. Written informed consents were obtained from all patients before undergoing any study-related procedures.

### Sample collection and cfDNA extraction

Whole blood samples (2 × 10 mL) from every patient were collected in cell-free DNA BCT tubes (Streck, USA). After centrifuging samples at 1600 g for 20 min at room temperature, the plasma supernatants were separated with blood cell sediment and then carefully transferred to new tubes, followed by re-centrifugation at 16,000 g for 10 min to remove the residual cells and debris. The buffy coat was then transferred to a new tube for genomic DNA (gDNA) extraction. Afterward, gDNA from white blood cells was extracted using DNeasy Blood & Tissue Kit (Qiagen, Germany) and cell-free DNA (cfDNA) from plasma was extracted using QIAamp Circulating Nucleic Acid Kit (Qiagen, Germany) following the manufacturer’s protocols. DNA was qualified with Nanodrop 2000 (Thermo Fisher Scientific), and quantified by Qubit 2.0 using the dsDNA HS Assay Kit (Life Technologies, USA) according to the manufacturer’s recommendations.

### Next-generation sequencing (NGS)

ctDNA sequencing was performed by 3D Medicines Inc, Shanghai, China. Before library construction, gDNA from white blood cells were sheared into 200–400 bp fragments using Covaris S2 Sonolab (Covaris, USA). Then, gDNA and cfDNA (30–60 ng) samples were processed with the KAPA Hyper Prep kit (KAPA Biosystems) for libraries construction according to the manual. An OncoKB-based NGS panel targeting 733 cancer-relevant genes (Table S2) or a customized NGS panel targeting 61 genes related to targeted therapy and immunotherapy for solid tumors (Table S3) was used for hybridization enrichment. Plasma cfDNA was labeled with unique molecular identifiers (UMI), enriched using targeted hybridization capture, and sequenced on the NovaSeq 6000 platform (Illumina) for 100 bp paired-end sequencing with a mean sequencing depth of 35000.

### Data processing and analysis

Quality control was performed with Trimmomatic. Raw data (fastq files) of paired samples were mapped to the reference human genome hg19 using Burrows-Wheeler Aligner (BWA-mem, v0.7.12). An in-house developed software was used to generate duplex consensus sequences based on dual UMI integrated at the end of the DNA fragments. The variant allele frequency (VAF), which reflects the altered ctDNA fraction among all cfDNA in plasma, was determined for all single-nucleotide variations (SNVs), insertions/deletions (indels) and copy number variations (CNVs). SNVs and indels were annotated by ANNOVAR against the following databases: dbSNP (v138), 1000 Genome, and ESP6500 (population frequency > 0.015). CNVs (gain/loss) were detected as described previously[28]. Potential germline variants and clonal hematopoietic variants were filtered out by analyzing genotyping results of gDNA extracted from paired white blood cells (see the principle of variants filtering in Supplementary Fig. 1 and the list of recurrent clonal hematopoietic variants in Table S5). To improve specificity, especially for variants with low allele frequency in the ctDNA, an in-house loci specific variant detection model based on a binomial test was applied. The variants were subsequently filtered by their supporting count, strand bias status, base quality, and mapping quality. The standard deviation of all segment level log-ratios was calculated for each sample, and segments with log-ratio above 3*standard deviation were defined as amplification.

The VAF was defined as the percentage of mutant reads in total reads. Concentration of ctDNA were expressed in nanogram per millilitre (ng/mL), determined as the product of total cfDNA concentration and the maximum VAF of somatic mutations, and expressed in base 10 log scale (log ng/mL). TMB (muts/Mbp) was calculated by summing all base substitutions and indels in the coding region of targeted genes, including synonymous alterations to reduce sampling noise and excluding known driver mutations as previously described [29].

### Statistical analysis

Differences between two groups were examined by the two-tailed, unpaired t test for normally distributed variables, whereas the Mann–Whitney test or Kruskal–Wallis test was used to assess the differences among two or three groups for non-normally distributed variables. The Fisher’s exact test was used to assess the differences of categorical variables between two groups. Progression-free survival (PFS) was measured from the date of treatment start to the date of disease progression or death from any cause or the last follow-up. Overall survival (OS) was measured from the date of diagnosis to the date of death from any cause and was censored at the date of the last follow-up. Survival analyses of PFS and OS were estimated using the Kaplan–Meier method with the Log-rank test, and the hazard ratio (HR) was calculated by Cox proportional hazards regression model. All reported *P* values were two-tailed, and *P* < 0.05 was considered as statistically significant. All statistical analyses were performed using SPSS software (v22.0; SPSS Inc., USA) or R program, and formatted with GraphPad Prism (v8.0; USA).

## Results

### Study design and patient characteristics

**Fig. 1:**
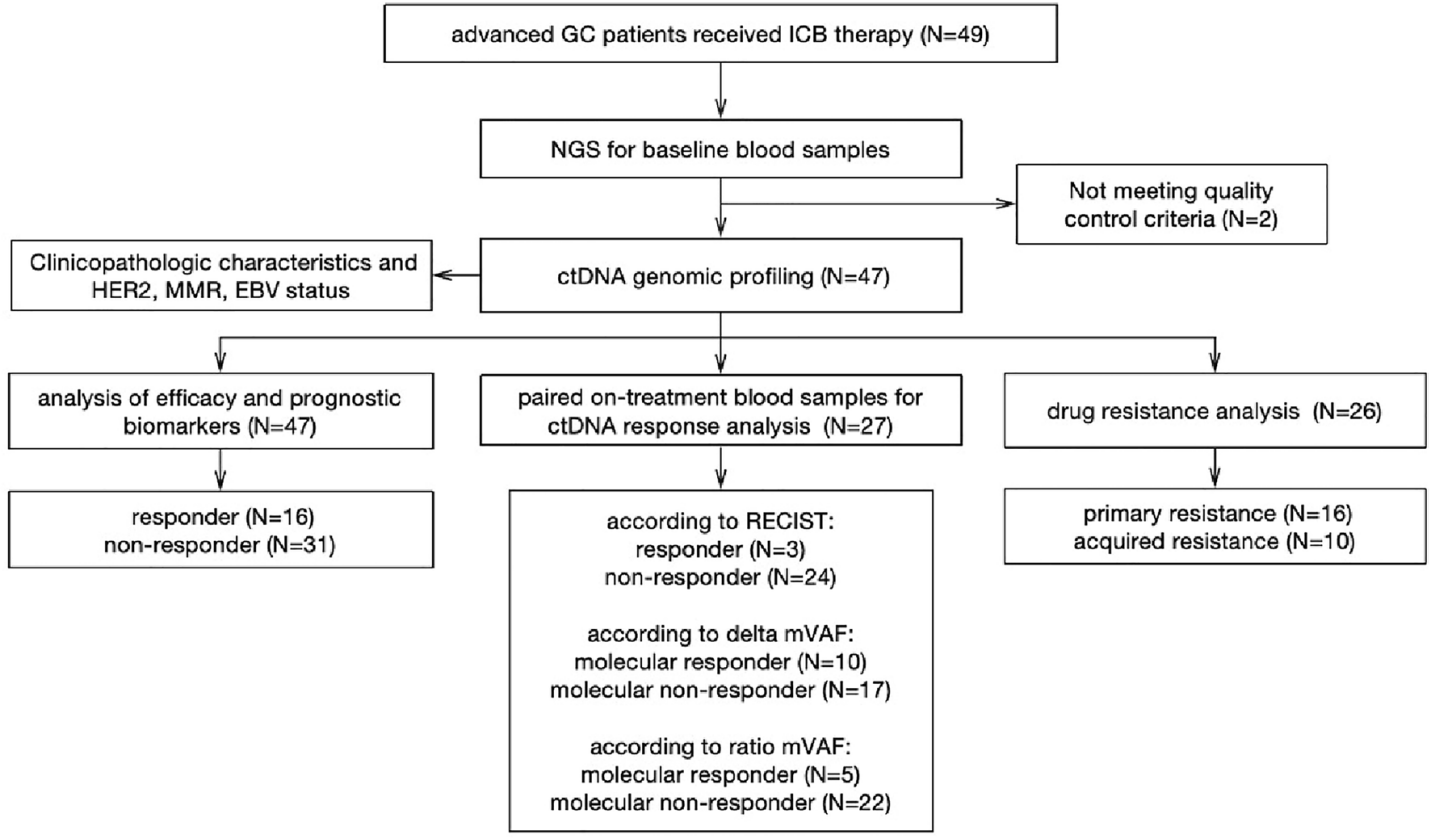
A diagram illustrating the study workflow and samples used for each step. *GC* gastric cancer, *NGS* next-generation sequencing, *ctDNA* circulating tumor DNA, *HER2* human epidermal growth factor receptor 2, *MMR* mismatch repair, *EBV* Epstein–Barr virus, *RECIST* response evaluation criteria in solid tumors, *mVAF* mean variant allele frequency

Forty-seven eligible GC patients received ICI or ICI plus chemotherapy were enrolled in this study between August of 2014 and February of 2022. The clinical and pathological characteristics are summarized in Table 1 and the procedure of data analysis was described in Fig. 1. The median age at diagnosis was 55 (range: 22–82) years, and the male: female ratio was 1.35:1. Patients predominantly had adenocarcinoma histology (89.4%) while other histologic subtypes included squamous cell carcinoma (4.3%), signet-ring cell carcinoma (4.3%) and mucinous adenocarcinoma (2.1%). Most patients were in clinical stage IV (95.7%) and were non-esophagogastric junction origination (72.3%), classified as intestinal (34.0%), or diffuse (31.9%), or mixed (21.3%) subtype. HER2 positivity was assessed using IHC or FISH, and all patients were HER2-negative. In fourty-six patients with available PD-L1, twenty-seven (57.4%) patients had tumors with PD-L1 CPS ≥ 1. Eight (17.0%) patients were dMMR/MSI-H and ten (21.3%) patients were confirmed to be EBV positive.

**Table 1:** Patient characteristics (N = 47)

Characteristics	Number	(%)
Median age (range)	55 (22-82)	
Gender		
Male	27	57.4%
Female	20	42.6%
Histologic subtypes		
Adenocarcinoma (including signet-ring cell carcinoma and mucinous adenocarcinoma)	45	95.7%
Squamous cell carcinoma	2	4.3%
Lauren classification		
Intestinal	16	34.0%
Diffuse	15	31.9%
Mixed	10	21.3%
NA	6	12.8%
Tumor stage		
III	2	4.3%
IV	45	95.7%
Tumor site		
EGJ	13	27.7%
NEGJ	34	72.3%
MSI status		
MSS	39	83.0%
MSI-H	8	17.0%
HER2 status		
HER2^−^	47	100.0%
PD-L1 status		
Negative	19	40.4%
Positive	27	57.4%
NA	1	2.1%
EBV status		
Negative	25	53.2%
Positive	10	21.3%
NA	12	25.5%
Therapy regimens		
Anti-PD-(L)1	18	38.3%
Anti-PD-(L)1 + anti-CTLA-4	12	25.5%
Anti-PD-(L)1 + chemotherapy	17	36.2%
Lines of treatment		
First-line	27	57.4%
Second-line	8	17.0%
Third-line	9	19.1%
Fourth-line	1	2.1%
Fifth-line	2	4.3%
Best overall response		
PR	16	34.0%
SD	16	34.0%
PD	15	31.9%

*EGJ* esophagogastric junction, *NEGJ* non-esophagogastric junction, *MSI* microsatellite instability, *MSS* microsatellite stable, *MSI-H* microsatellite instability high, *HER2* human epidermal growth factor receptor 2, *PD-L1* programmed death ligand 1, *EBV* Epstein–Barr virus, *PR* partial response, *SD* stable disease, *PD* progressive disease, *NA* not available

More than one third (36.2%) of the patients received anti-PD-(L)1 treatment plus chemotherapy as first-line therapy, while others received anti-PD-(L)1 (38.3%) or anti-PD-(L)1 plus anti-CTLA-4 treatment (25.5%) as first-line or late line therapy. The PD-1 antibody included Nivolumab, Pembrolizumab, Toripalimab, Sintilimab, Tislelizumab, Zimberelimab, Camrelizumab and LZM009. The PD-L1 antibodies included Durvalumab, Atezolizumab, Envafolimab, Sugemalimab and MSB2311. The CTLA-4 antibodies included Ipilimumab, Tremelimumab, IBI310 and bispecific PD-1/CTLA-4 antibody Cadonilimab. The combination chemotherapy regimens or drugs employed include XELOX (capecitabine plus oxaliplatin), SOX (tegafur plus oxaliplatin), and PTX. The median follow-up time was 10.03 months. Response evaluations were available for all patients, and the BOR was defined as the best response documented from initiation of treatment until the end of treatment. The median follow-up for BOR was 1.47 months. Specifically, sixteen (34.0%) patient achieved PR, sixteen (34.0%) had SD, and fifteen (31.9%) patients were PD, whereas no CR was observed in this cohort. The detailed clinicopathological information of all patients and specimens were concluded in Table S1.

### Mutational profile assessed by baseline ctDNA

**Fig. 2:**
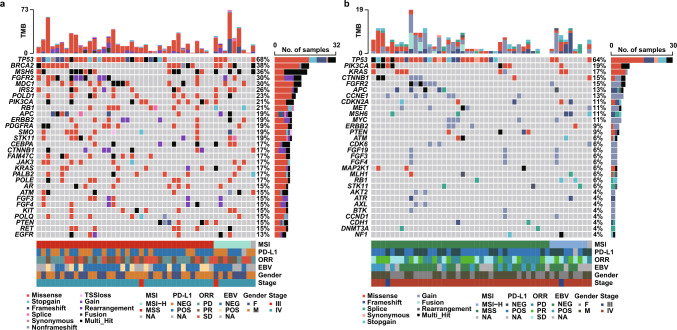
The landscape of high-frequency genomic alterations detected in 47 baseline blood samples. Oncoplot of somatic mutations containing variants of unknown clinical significance (**a**) or only pathogenic variants (**b**) detected in the cohort using a 733-gene panel. Somatic variants are classified and interpreted according to AMP/ASCO/CAP standards and guidelines. Each column represents one sample and each row represents one gene. The upper bars represent the tumor mutational burden (TMB) in each patient. The right bars represent the numbers of patients for specific genes in the total cohort and the corresponding proportions are listed on the side. Cases are clustered according to MSI status. PD-L1 status, ORR, EBV status, gender, and tumor stage are also shown

Plasma ctDNA sequencing results were obtained using an OncoKB-based 733-gene panel (Table S2). A total of 658 somatic mutations involving 203 genes were identified among these 47 patients with HER2-negative advanced GC. The median number of mutations per sample was 11 (range: 0–73) with a mean variant allele frequency (mVAF) of 5.3% (range: 0.0–27.0%) and the maximum VAF of 85.3%. Figure 2 shows the mutational profile revealed from the baseline samples, restricted to genes mutated in more than 4% of the cases including pathogenic mutations and mutations of unknown clinical significance. The most frequently mutated genes revealed by ctDNA genotyping were *TP53* in 68% of cases, *BRCA2* in 38%, *MSH6* in 36%, *FGFR2* and *MDC1* in 30% of cases (Fig. 2a). Among the pathogenic mutations, *TP53* (64%), *PIK3CA* (19%), and *KRAS* (17%) were the most frequent gene alterations (Fig. 2b).

### Correlation between pretreatment ctDNA and baseline clinical factors

**Fig. 3:**
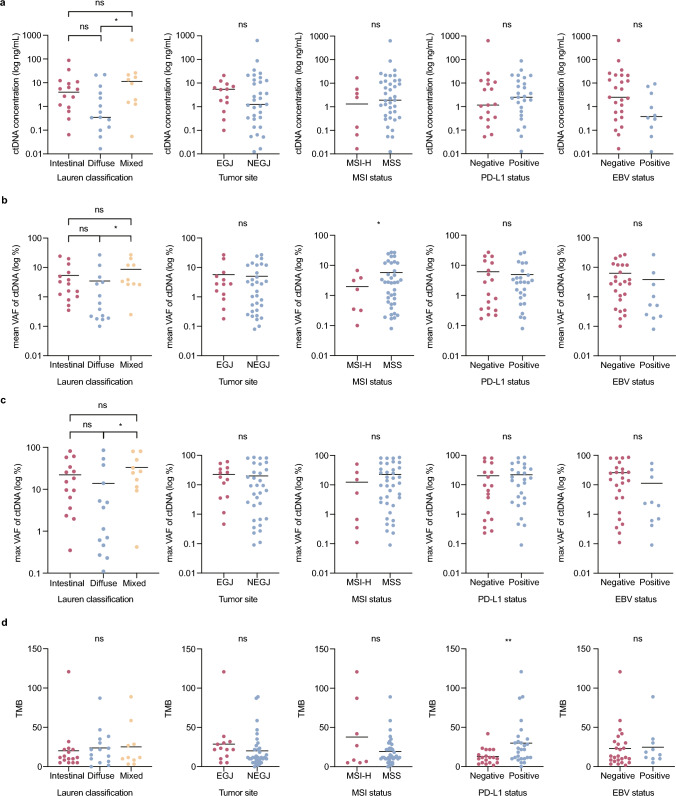
Correlation between pretreatment ctDNA parameters and baseline clinical factors. Comparison of pretreatment ctDNA concentration (**a**), the mean VAF (**b**), maximum VAF (**c**), and TMB (**d**) detected in pretreatment cfDNA between Lauren classification (Intestinal N = 16, Diffuse N = 15, Mixed N = 10), tumor site (EGJ N = 13, NEGJ N = 34), MSI status (MSI-H N = 8, MSS N = 39), PD-L1 status (Negative N = 19, Positive N = 27) and EBV status (Negative N = 25, Positive N = 10). When using the logarithmic axis in (**a–c**), 2 cases with a value of zero are not plotted. The horizontal line represents the mean of each group. Kruskal–Wallis test was used to assess the differences among patients with different Lauren classification. Two-tailed unpaired t test with Welch’s correction was used to assess the differences among patients with different tumor site, MSI status, PD-L1 status and EBV status. *P value < 0.05; **P value < 0.01; *ns* not significant

We further compared pretreatment ctDNA parameters, including the ctDNA concentration, mVAF, maximum VAF and TMB detected, between patients grouped by different clinical factors including Lauren classification, tumor site, and MSI/PD-L1/EBV status. The mean concentration of pretreatment ctDNA was 20.9 (range: 0–631.4) ng/mL. The levels of pretreatment ctDNA concentration, mVAF and maximum VAF were higher in patients with mixed type of GC rather than diffuse type (*P* < 0.05). However, there was no significant difference between these parameters between intestinal and mixed or diffuse types (Fig. 3a–c). The mVAF was higher in patients with MSS GC compared to MSI-H (*P* < 0.05, Fig. 3b), and the TMB of patients who had a positive PD-L1 status was significantly higher than those who did not (*P* < 0.01) (Fig. 3d).

### Baseline ctDNA gene alterations as biomarkers of ICB efficacy

**Fig. 4:**
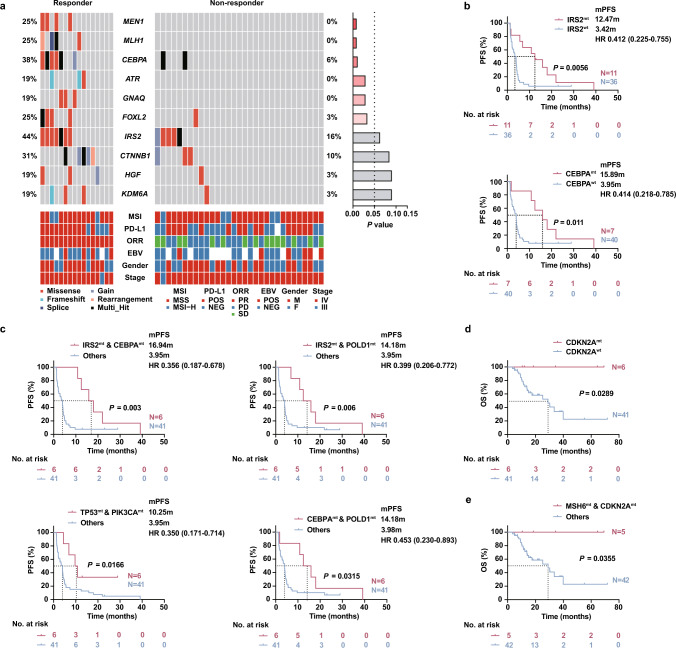
Mutational status of specific genes in baseline ctDNA predicted the clinical outcomes of ICB treatment. **a** Oncoplot of the top 10 differential baseline mutations in the responders versus non-responders. Responder refers to patients who achieved PR for best overall response (N = 16); non-responder refers to patients who achieved SD/PD for best overall response (N = 31). Each column represents one sample and each row represents one gene. The proportions of patients for specific genes in each cohort are listed on the side. MSI status, PD-L1 status, ORR, EBV status, gender, and tumor stage are shown by cases. The right bars show *P* values by Fisher’s exact test. **b** Progression-free survival (PFS) for patients with different IRS2 or CEBPA mutation status in baseline plasma ctDNA. **c** PFS for patients with different co-occurring mutation status including *IRS2* & *CEBPA*, *IRS2* & *POLD1*, *TP53* & *PIK3CA*, *POLD1* & *CEBPA* in baseline plasma ctDNA. **d** Overall survival (OS) for patients with different *CDKN2A* mutation status in baseline plasma ctDNA. **e** OS for patients with different co-occurring *MSH6* & *CDKN2A* mutation status in baseline plasma ctDNA. Survival proportions were assessed by Kaplan–Meier survival analysis paired with Log-rank test. HR: hazard ratio, 95% confidence interval was shown and not available in **d** and **e** because nobody died in the mutant groups; *mt* mutant, *wt* wild-type

Besides describing the mutational landscape of HER2-negative GC at advanced stage, we aimed at assessing whether specific mutations detected in ctDNA correlate with response to ICB treatment. For this, the 47 GC patients were divided into two groups based on their clinical response to the treatment. Sixteen patients with the BOR achieved PR were categorized into the responder group, while thirty-one patients achieved SD/PD were categorized into the non-responder group. Of the top ten differentially mutated genes in responders versus non-responders, six genes were found to be more likely to mutate in responders. These genes include *MEN1* (*P* = 0.01), *MLH1* (*P* = 0.01), *CEBPA* (*P* = 0.01), *ATR* (*P* = 0.03), *GNAQ* (*P* = 0.03), and *FOXL2* (*P* = 0.04) (Fig. 4a).

Furthermore, we conducted a survival analysis and observed a significant extension of median PFS (mPFS) in *IRS2*-mutant (12.47 vs. 3.42 months; log rank* P* = 0.0056; HR = 0.412; 95% CI 0.225–0.755) or *CEBPA*-mutant (15.89 vs. 3.95 months; *P* = 0.011; HR = 0.414; 95% CI 0.218–0.785) patients compared with wild-type patients (Fig. 4b, Table S4). Then, we analyzed the association of co-occurring genetic alterations with survival. Patients with both *IRS2* and *CEBPA* mutations in their baseline plasma samples had a significantly better mPFS than the others (16.94 vs. 3.95 months; *P* = 0.003; HR = 0.356; 95% CI 0.187–0.678; Fig. 3c, Table S4). In addition, the presence of *IRS*2 & *POLD1* (14.18 vs. 3.95 months; *P* = 0.006; HR = 0.399; 95% CI 0.206–0.772), *CEBPA* & *POLD1* (14.18 vs. 3.98 months; *P* = 0.0315; HR = 0.453; 95% CI 0.230–0.893), *TP53* & *PIK3CA* (10.25 vs. 3.95 months; *P* = 0.0166; HR = 0.350; 95% CI 0.171–0.714) mutations also predicted better mPFS than the others (Fig. 4c, Table S4). Moreover, both alteration of *CDKN2A* alone (*P* = 0.0289; Fig. 4d, Table S4) and co-mutations with *MSH6* (*P* = 0.0355; Fig. 4e, Table S4) were significantly associated with superior OS. Based on our findings, it is estimated that alterations in the indicated genes might indicate a good response to ICB therapy in patients with advanced GC.

### Decreasing ctDNA was correlated with higher response to ICB

**Fig. 5:**
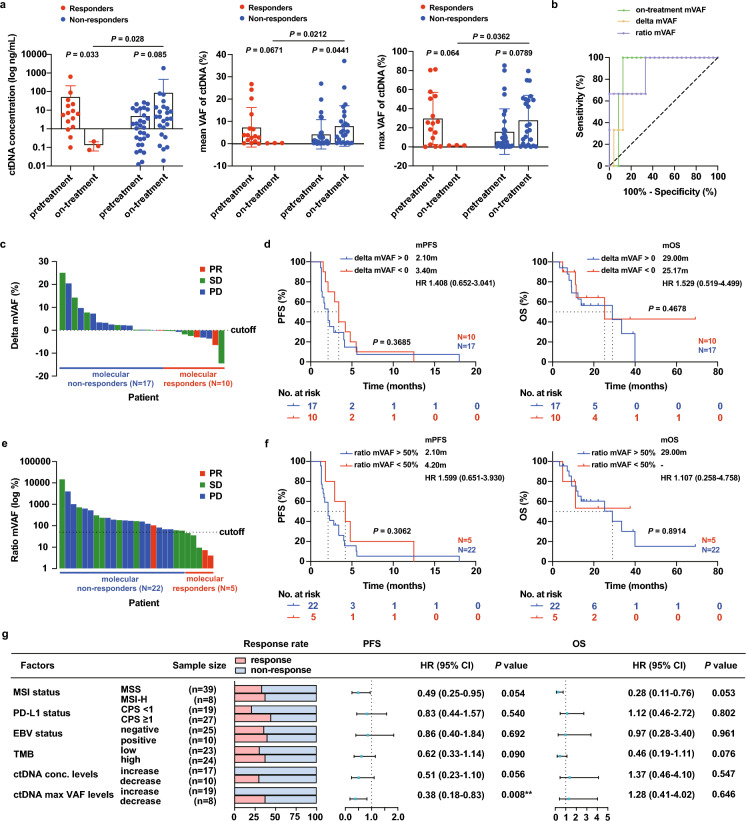
CtDNA dynamics are predictive of clinical benefits from immune checkpoint blockade. **a** Comparison of the ctDNA concentration, mean VAF and maximum VAF between pre-treatment and on-treatment for the responders and non-responders. Responder refers to patients who achieved PR for best overall response (pre-treatment N = 16, on-treatment N = 3); non-responder refers to patients who achieved SD/PD for best overall response (pre-treatment N = 31, on-treatment N = 24). Mann–Whitney test was used to assess the differences between pre-treatment and on-treatment samples, or responders and non-responders. **b** ROC curves of molecular response and on-treatment mVAF to predict the best response. Waterfall plot of delta mVAF (**c**) or ratio mVAF (**e**) levels in descending order for individual patients, color coded by clinical responses evaluated by CT scan. The dotted line shows the cutoff of the molecular response as defined by either delta mVAF < 0 (**c**) or ratio mVAF < 50% (**e**). Kaplan–Meier estimates of PFS (left) and OS (right) according to delta mVAF (**d**) or ratio mVAF (**f**) levels. **g** Response rate, and forest plot for PFS and OS, based on different prognostic factors. The mutation load to classify TMB into high and low level was top 25% (cutoff = 13 mutations). Survival proportions were assessed by Kaplan–Meier survival analysis paired with Log-rank test. *Conc.* Concentration, *HR* hazard ratio; horizontal lines show the 95% confidence interval (CI) for each factor

The change in ctDNA immediately post-treatment has been reported to predict response to PD-(L)1 inhibition in solid tumors[6, 30, 31]. To test this hypothesis, dynamic sampling was performed at the on-treatment timepoint of average 8.3 weeks in 27 patients, varying between 3.9–19.1 weeks from the start of treatment. Among the responding patients, ctDNA concentrations decreased significantly at the on-treatment timepoint (*P* = 0.033), as did mVAF and max VAF levels, although not reaching statistical significance in the latter two parameters (Fig. 5a). Among the non-responding patients, an opposite trend could be observed in the indicated parameters. For example, a significant increase in mVAF was observed in non-responders from pretreatment to the on-treatment timepoint (*P* = 0.0441; Fig. 5a). Moreover, higher ctDNA concentration/mVAF/max VAF at the on-treatment time point were associated with poorer response (responders vs. non-responders, *P* = 0.028, 0.012, 0.0362 respectively; Fig. 5a). Taken together, these results suggest that dynamic changes in ctDNA and on-treatment ctDNA levels may be predictive of response to treatment with ICIs.

The short half-life of ctDNA offers a unique opportunity to utilize early on-treatment changes in ctDNA for real-time assessment of therapeutic response and outcome, termed molecular response [3]. As both the change in ctDNA and on-treatment ctDNA level were notably associated with efficacy of ICB therapy, we further analyzed the predictive effect of the molecular response determined by integrating information from pretreatment and on-treatment VAF. Using receiver operating characteristic (ROC) analysis, we verified that the ratio-based and delta-based molecular response metric had similar association with RECIST response compared with on-treatment VAF alone (AUC = 0.89; 95% CI 0.69–1.00 for ratio mVAF; AUC = 0.83; 95% CI 0.65–1.00 for delta mVAF; AUC = 0.90; 95% CI 0.79–1.00 for on-treatment mVAF; Fig. 5b). Patients with pretreatment and on-treatment samples in the cohort were stratified into ctDNA-defined molecular responders and non-responders by a cutoff point of 0 in delta mVAF or of 50% in ratio mVAF, consistent with previous works[32–35]. Molecular responders stratified by delta mVAF had higher objective response rate (ORR) than molecular non-responders (20.0% vs. 5.88%, *P* = 0.535; Fig. 5c), with slightly longer mPFS (Fig. 4d). Stratification of the same cases by ratio mVAF demonstrated similar findings in ORR (40.0% vs. 4.55%, *P* = 0.0786; Fig. 5e) and a trend toward improved mPFS and median OS (Fig. 5f) in molecular responders.

In univariate analysis, established prognostic factors for immunotherapy including MSI status, PD-L1 status, EBV status, and TMB along with ctDNA kinetic parameters were incorporated. The data show that changes in ctDNA concentration levels and max VAF levels could be better indicators of treatment response than other established factors. Univariate analysis found that a reduction in ctDNA max VAF levels were associated with better PFS (*P* = 0.008; HR = 0.38; 95% CI 0.18–0.83; Fig. 5g). These findings suggest that ctDNA kinetics has great potential in capturing the clinical outcomes of ICB treatment in patients with advanced GC.

### Changes in ctDNA predict resistance to ICB

**Fig. 6:**
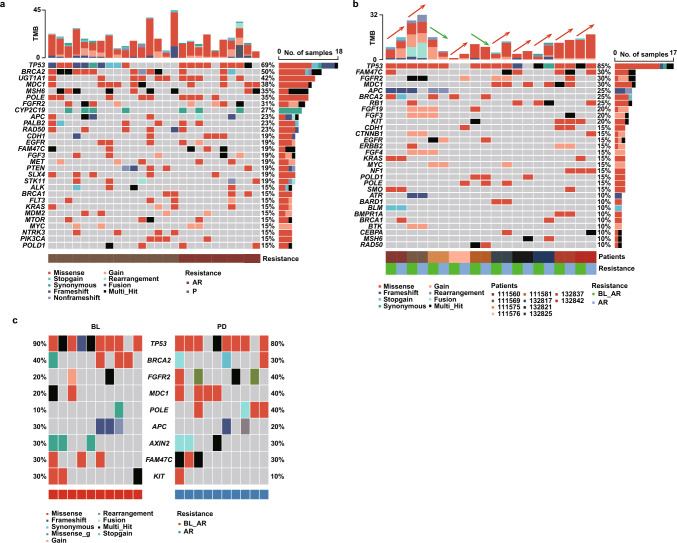
Molecular monitoring of ctDNA upon ICB treatment. **a** Heatmap of mutation profiles at the time of progression for resistant patients receiving ICB therapy according to response to treatment (N = 26). Each column represents one sample and each row represents one gene. The upper bars represent the TMB in each patient. **b** Mutation profiling of ctDNA before and at progression during ICB treatment for ten patients who progressed during follow-up. The two adjacent columns represent the baseline and on-progression data for the same patient pairing. The upper bars represent the change in TMB of each patient. The right bars represent the numbers of patients for specific genes in the subgroup and the corresponding proportions are listed on the side. **c** Comparison of molecular alterations at baseline and PD in acquired resistant patients. Each column represents one sample and the corresponding column matched to the same patient. The proportions of patients for specific genes in each cohort are listed on the side. *AR* acquired resistance (N = 10); *P* primary resistance (N = 16); *BL* baseline, *PD* progressive disease

GC harbours intensive intrinsic molecular heterogeneity, which might be a critical driver of the resistance to immunotherapy. ctDNA provides good opportunities for longitudinal analysis which could be employed as a real-time tool for monitoring of emergent resistance mutations in patients receiving ICB therapy. Throughout the whole follow-up period, 43 patients had progressed with ICB therapies (mPFS = 3.98 months, range: 1.15–39.11 months). 26 of these progressed cases had available plasma samples at the time of progression, of which 10 cases developed acquired resistance (mPFS = 3.98 months) and 16 cases developed primary resistance (mPFS = 1.46 months). Generally, considerable consistency was present in the high-frequency mutations detected in the resistant population at PD and the total population at baseline, with *TP53* and *BRCA2* mutations ranked the top two (69% and 50% at PD, 68% and 38% at baseline, respectively; Figs. 2a and 6a). The proportion of mutations in most of these high-frequency mutant genes increased at PD in the resistant patients, including an 18% increase in *POLE*, 12% increase in *BRCA2* and 8% increase in *MDC1*, ranked the top three.

To explore the acquired resistance of ICB treatment, we compared 10 available plasma samples at PD of the patients who had benefit from the treatment (achieved PR or SD) with paired baseline samples. Most of the TMB at PD were to varying degrees increased compared with baseline, and new genomic alterations were observed in 8 patients (Fig. 6b). Mutations of *POLE* gene were identified as new alterations in 3 patients, and *BRCA1*/*FGFR2*/*CEBPA*/*MSH6* mutations were also found as new alterations in 2 patients. Other emerging genes found in one patient include *BRCA2*, *EGFR*, *MYC*, *MDC1*, etc. Specifically, the molecular alterations of *POLE*, *FGFR2* and *MDC1* were more frequent at PD than baseline in patients with acquired resistance (40% vs. 10%, 40% vs. 20%, 40% vs. 20%, respectively; Fig. 6c). These findings suggest that alterations in these genes may contributed to acquired resistance to ICB treatment.

## Discussion

Using mutation profiling of ctDNA, we have provided a comprehensive characterization of the mutational landscape of patients with HER2-negative advanced GC. This approach, utilizing liquid biopsy, offers significant advantages over traditional tissue specimen analysis, including minimally invasive collection, convenience, and the ability to capture the complexity of intra- and inter-tumor heterogeneity [3, 36, 37]. It has been reported that the sensitivity of ctDNA in detecting tissue-confirmed mutations ranges from 60% to 80% in GC, depending on the detection technique used and the characteristics of the sample. Although mutation profiling from paired tissue samples were lacking in this study, most of the common mutated genes such as *TP53*, *APC*, *KRAS*, *PIK3CA*, *MSH6*, *ATR* etc., were detectable, compared to previous reports using tissue samples [38, 39]. In particular, we identified some specific gene alterations associated with treatment response, survival, and tumor progression.

Our analysis revealed that missense mutations in the *MEN1* gene were significantly associated with enhanced responses to ICB therapies. Given the established role of *MEN1* as a tumor suppressor, these mutations may indicate a potential link to increased immunogenicity or altered signaling pathways that sensitize tumors to ICB [40]. Additionally, patients harboring mutations in *CEBPA* were correlated with better response and exhibited a substantially longer mPFS, indicating the potential of *CEBPA* as a strong predictive marker for responsiveness to ICB therapies. This aligns with existing literature indicating that *CEBPA* mutations may facilitate immune cell recruitment and tumor microenvironment remodeling, thereby influencing prognosis across various cancer types [41, 42]. Furthermore, mutations in *IRS2* were associated with extended PFS, highlighting its role in modulating cellular responses to immunotherapy, particularly through the PI3K/AKT signaling pathway, which is implicated in immune escape mechanisms [43, 44]. The study also identified co-occurring mutations, such as those in *IRS2*, *CEBPA*, and *POLD1*, which demonstrated significant correlations with better PFS outcomes, underscoring the multifactorial nature of biomarker development.

The rapid progression of cancer patients may be due to acquired resistance mutations and pre-existing tumor molecular heterogeneity, while ctDNA testing has the potential to uncover underlying resistance mechanisms. However, the interactions among these genetic alterations and their contribution to immunotherapy resistance are complex. Notably, mutations in DNA polymerase genes (*POLE* and *POLD1*) and genes involved in DNA damage repair pathways (e.g., *BRCA2*, *MSH6*) were linked to high TMB, which is associated with immunotherapy responsiveness but may also contribute to drug resistance [45–48]. Additionally, inactivation of *MDC1*, a mediator of DNA damage checkpoints, may further impact the efficacy of immunotherapy [49, 50]. Together, these evidences suggest that the above genes are expected to serve as promising potential predictive biomarkers for ICB treatment and that the underlying contribution of these genes to resistance needs to be further explored.

Multiple studies have correlated early on-treatment ctDNA changes with response to various immunotherapies across several advanced solid tumors [6, 30, 35, 51]. Our study investigates the use of ctDNA dynamics to predict the clinical efficacy of ICB therapies in HER2-negative advanced GC patients. Key findings indicated that significant decreases in on-treatment ctDNA concentrations were associated with responders, while increases correlated with non-responders. Higher levels of on-treatment ctDNA were inversely related to treatment response, suggesting that patients with elevated tumor burdens may exhibit more aggressive disease biology or tumor heterogeneity, leading to poorer outcomes. Additionally, the two mainstream calculating methods of molecular response appear to have similar prediction validity in this study, and more samples are needed for further comparison and optimization.

Despite the promising results, our study has limitations, including its retrospective design and the small sample size, which may introduce statistical bias. Additionally, concurrent tissue sequencing was lacking, restricting our ability to fully assess the sensitivity and specificity of ctDNA NGS methods. The findings presented herein may serve as a foundation for future large-scale, multicenter prospective studies.

The utilization of ctDNA as a predictive biomarker holds significant promise in the realm of personalized medicine, especially in unresectable or metastatic GC where treatment options remain limited. Our study retrospectively analyzed ctDNA from plasma samples of HER2-negative advanced GC patients who underwent immune checkpoint blockade therapies, revealing critical variations associated with treatment response, offering insights that could enhance the stratification of patients for more effective interventions.

## Supplementary Information

Below is the link to the electronic supplementary material.

Supplementary file1 Supplementary Fig. 1 ctDNA filtering principles and variant filtering statistics for each sample. a The principle of genetic variants filtering for ctDNA used in this study: variants to be confirmed are first filtered out if they are included in the list of recurrent CHIP variants. Otherwise, if they exist in the WBC sample and meet the criteria for positivity (VAF ≥ 0.05), take the next step: (1) If the genotypes are homozygous or heterozygous, they are considered as germline variants; (2) If they do not satisfy homozygosity or heterozygosity, and also the Fisher’s exact test is significant, they are considered as ctDNA contamination; 3) If they do not satisfy homozygosity or heterozygosity, and also the Fisher’s exact test is not significant, they are considered as CHIP variants. b The number of variants filtered out and the number of variants retained in each of the 47 patients as a percentage of the total number of variants.(PDF 471 KB)

Supplementary file2 (XLSX 45 KB)

## Data Availability

The datasets used in this study are available from the corresponding authors on reasonable request.
