# Supplementary figures and images for: Circulating tumor DNA predicts clinical benefits of immune checkpoint blockade in HER2-negative patients with advanced gastric cancer
Source: Gastric Cancer. 2025 May 15;28(5):872–85. doi: 10.1007/s10120-025-01621-x (PMC12378147; doi:10.1007/s10120-025-01621-x)

**Supplementary Fig.1**

**a**

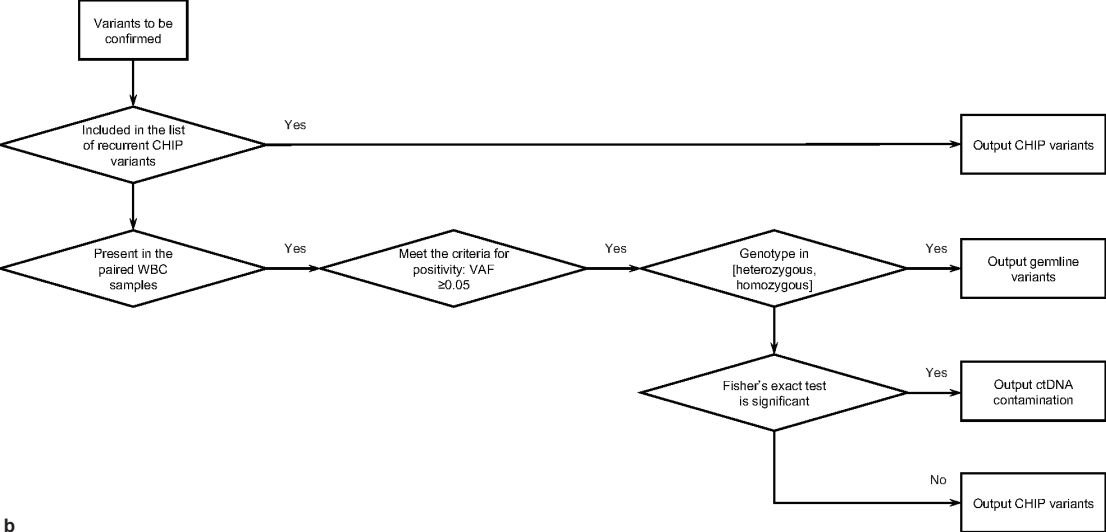

**b**

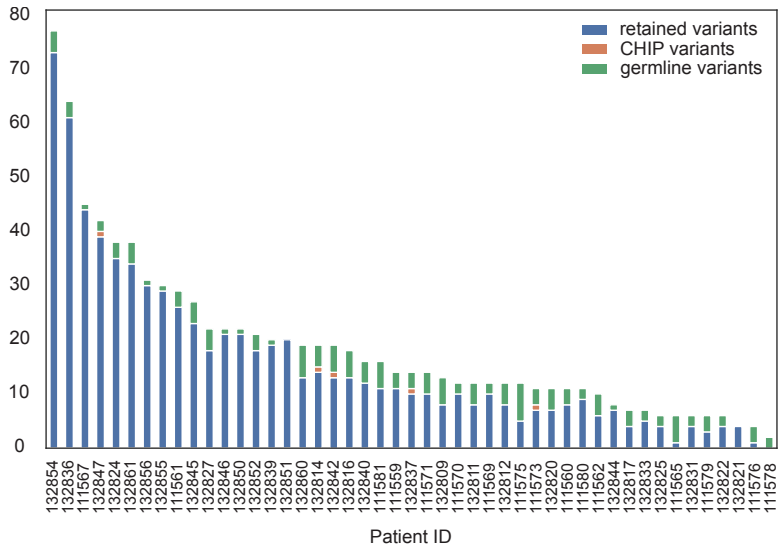

Supplement: Supplementary file 1 — Supplementary file1 Supplementary Fig. 1 ctDNA filtering principles and variant filtering statistics for each sample. a The principle of genetic variants filtering for ctDNA used in this study: variants to be confirmed are first filtered out if they are included in the list of recurrent CHIP variants. Otherwise, if they exist in the WBC sample and meet the criteria for positivity (VAF ≥ 0.05), take the next step: (1) If the genotypes are homozygous or heterozygous, they are considered as germline variants; (2) If they do not satisfy homozygosity or heterozygosity, and also the Fisher’s exact test is significant, they are considered as ctDNA contamination; 3) If they do not satisfy homozygosity or heterozygosity, and also the Fisher’s exact test is not significant, they are considered as CHIP variants. b The number of variants filtered out and the number of variants retained in each of the 47 patients as a percentage of the total number of variants.(PDF 471 KB) [file 10120_2025_1621_MOESM1_ESM.pdf]
